# Animal Models of Uveal Melanoma: Methods, Applicability, and Limitations

**DOI:** 10.1155/2016/4521807

**Published:** 2016-06-06

**Authors:** Marta M. Stei, Karin U. Loeffler, Frank G. Holz, Martina C. Herwig

**Affiliations:** Department of Ophthalmology, University of Bonn, Ernst-Abbe-Straße 2, 53127 Bonn, Germany

## Abstract

Animal models serve as powerful tools for investigating the pathobiology of cancer, identifying relevant pathways, and developing novel therapeutic agents. They have facilitated rapid scientific progress in many tumor entities. However, for establishing a powerful animal model of uveal melanoma fundamental challenges remain. To date, no animal model offers specific genetic attributes as well as histologic, immunologic, and metastatic features of uveal melanoma. Syngeneic models with intraocular injection of cutaneous melanoma cells may suit best for investigating immunologic/tumor biology aspects. However, differences between cutaneous and uveal melanoma regarding genetics and metastasis remain problematic. Human xenograft models are widely used for evaluating novel therapeutics but require immunosuppression to allow tumor growth. New approaches aim to establish transgenic mouse models of spontaneous uveal melanoma which recently provided preliminary promising results. Each model provides certain benefits and may render them suitable for answering a respective scientific question. However, all existing models also exhibit relevant limitations which may have led to delayed research progress. Despite refined therapeutic options for the primary ocular tumor, patients' prognosis has not improved since the 1970s. Basic research needs to further focus on a refinement of a potent animal model which mimics uveal melanoma specific mechanisms of progression and metastasis. This review will summarise and interpret existing animal models of uveal melanoma including recent advances in the field.

## 1. Introduction

Animal models of human cancer can contribute to the understanding of cancer pathobiology and the development of novel therapeutic agents. The ultimate goal is to translate scientific progress from basic research (*in vitro *and* in vivo*) through preclinical animal studies finally into human clinical trials to demonstrate both efficacy and safety. However, the absence of effective* in vivo* systems that accurately mimic the human disease condition and reliably predict clinical efficacy has hindered therapy and drug development in oncology [1]. Despite a poor rate of successful translation from animal models to clinical cancer trials [2],* in vivo* models have revolutionized options to study tumor biology and better understand molecular and genetic pathways. Cancer xenografts and genetically engineered mice are the most commonly used cancer models of several tumor entities [3]. In skin melanoma, genetically engineered mouse models revealed to be an improved prediction model of anticancer therapeutics and patients' response [4]. Such transgenic mouse models have been developed for many different tumor entities allowing for detailed and diverse studies for basic research purposes as well as preclinical drug screening. However, in some tumor entities like uveal melanoma fundamental challenges in establishing an animal model which meets the tumor's specific characteristics have not been overcome yet.

Uveal melanoma is the second most common type of melanoma after cutaneous and has, compared to skin melanoma, a relatively low incidence (6 per million per year, in the US [5]). However, it represents the most frequent primary intraocular tumor in the adult Caucasian population [6–8]. It arises from intraocular melanocytes of the choroid, the ciliary body, and/or the iris. Treatment strategies comprise brachytherapy, surgical excision, and removal of the entire globe. Even if fresh tumor material is available, establishing cell lines from uveal melanoma remains difficult and is often unsuccessful. There are only a limited number of permanent cell lines for uveal melanoma research available. Most of these were established some time ago which may have led to alterations in biologic and genetic properties. Thus, there is an urgent need for an effective animal model of uveal melanoma. Such a model should accurately mimic different characteristics of uveal melanoma such as genetics (monosomy 3, GNAQ/GNA11, and BAP1 mutations), hematogenous spread to the liver, (as the eye lacks lymphatics), an inflammatory tumor microenvironment, and other tumor growth characteristics. Intraocular melanomas have rarely been described in companion animals like dogs [9–11] and cats [12–15] or in other animals like horses [16], cattle [17], or others. Despite arising naturally, these intraocular melanomas occurred sporadically and unpredictably and mostly did not metastasize to distant organs. Thus, they do not qualify as an animal model for experimental uveal melanoma. Unfortunately, no spontaneous uveal melanoma has been observed in wild type mice to date. However, mice are the most commonly employed animal model in uveal melanoma studies due to the uncomplicated housing, availability, and genetic manipulation options. It is commonly accepted that, besides transgenic models, iatrogenically induced tumor models represent the best option for oncology research. This includes implantation of animal and human uveal melanoma cell lines into animals to model the behavior of this tumor. Since advanced age is associated with changes in immunologic constitution and inoculated tumors arising in old mice better resemble tumors of their human counterpart, generally, old mice should preferably be used for studies on tumor biology (Stei et al., unpublished data).

To generate and study intraocular melanoma in mice, basically four types of models have been developed including (1) intraocular inoculation with cutaneous melanoma cells into wild type mice, (2) injection of human uveal melanoma cells into mice which requires immunosuppression to allow tumor growth, (3) new approaches aiming to crossbreed or generate genetically engineered mice which spontaneously develop intraocular melanomas, and (4) induction of uveal proliferation by chemicals, radiation, or viruses [18]. Further, in order to study metastatic uveal melanoma, different models of hepatic metastases have been established. Animal models of uveal melanoma other than mice include chick embryos, drosophila, zebrafish, rats, and rabbits. However, as no optimal animal model that faithfully replicates the behavior of the human disease (spontaneously occurring and concurrently metastasizing) has been described yet, all established animal models represent compromises and are facing certain limitations.

This review will summarise and interpret the different types of existing animal models of uveal melanoma including recent advances in the field.

## 2. Animal Models of Uveal Melanoma

### 2.1. Intraocular Injection of Cutaneous Melanoma Cells

Syngeneic transplantation models are useful, especially for experiments designed to study or modulate immune responses which require an intact immune system. For cutaneous melanoma, the most frequently used syngeneic murine model is the B16 melanoma which was derived from a spontaneously arising melanoma in a C57Bl/6J mouse and was then established as a permanent cell line [19]. Accordingly, an initial approach to create a model of intraocular melanoma was to inject B16 cutaneous melanoma cells into the eyes of C57Bl/6 mice by a microinjection technique. Different sublines of B16 cells (F10, LS9, etc.) with different metastatic rates and other cutaneous melanoma cell lines such as HCmel12 have been employed for this purpose [20–23]. These cell lines form solid intraocular melanomas with characteristic tumor growth properties which would qualify as a model of intraocular melanoma. Further, the B16LS9 cell line has been selectively developed after serial passages for liver specific metastasis leading to the only model with an ocular tumor metastasizing to the liver [22]. These syngeneic mouse models have since been used in numerous studies mainly to investigate immunologic and angiogenic aspects but also to investigate tumor progression and imaging methodology [20, 21, 24–37]. Besides in mice models, inoculation with cutaneous melanoma cells has also been established in other rodents. Early oncologic experiments have been performed in hamsters by implanting the Greene (actually of rabbit origin) and later the Bomirski melanoma cell line into the anterior chamber [38–41]. Rabbit models provide the advantage of larger eyes and thus allow for an easier examination of experimental intraocular melanoma. The Greene melanoma cell line was implanted into the anterior or posterior chamber as well as into the subchoroidal space of rabbits and was formerly more commonly used for evaluation of treatment effects on intraocular melanomas [18, 42, 43]. However, rapid tumor growth and other complications have repelled this model. Approaches of grafting murine B16F10 cells into rabbit eyes resulted indeed in solid ocular tumors but metastases were not reliably observed and immune suppression was necessary which represented a disadvantage of this model [44, 45].

However, advantages of these syngeneic models include an apparent resemblance of the intraocular cutaneous melanoma compared to human uveal melanomas and the reliable reproducibility of this technique. The melanoma inside the unique intraocular microenvironment can be investigated in an immunologically intact mouse or other hosts. However, the application of different cell lines and different inoculation sites (anterior chamber, posterior chamber, intravitreal, subretinal, or retroorbital injection) complicates the comparability of the reported results. Furthermore and most importantly, one needs to keep in mind underlying genetic differences between cutaneous and uveal melanoma cells. Unlike cutaneous or conjunctival melanoma, mutations in B-RAF, RAS, or KIT genes occur rarely in uveal melanoma [46]. Characteristic mutations differ between uveal and cutaneous melanoma and even among tumors itself, accounting for different progression and metastatic behavior [47]. Although results from these models are suitable to describe immune responses and intraocular tumor behavior they need to be interpreted with caution when translating respective findings into treatment efficacy in human uveal melanoma.

### 2.2. Injections of Human Uveal Melanoma Cells

The development of xenograft models was an important step in improving animal models of clinical efficacy [95]. In cutaneous melanoma as well as in other tumor entities, human tumor xenograft models are widely used for drug screening, to evaluate targeted therapies or to test combinatorial efficacy of therapeutic agents [96]. For evaluation of intraocular melanoma growth and treatment strategies human xenografts (human uveal melanoma cell lines or primary tumor fragments) are commonly examined and several models have been described. Permanent cells lines grown from human uveal melanomas can be characterized histologically and by genetic profiling. They offer the opportunity to investigate biological and pharmacological aspects* in vitro* or in an animal model. Generally, these xenografts are inoculated into the eye of an immunosuppressed animal or in few cases they are injected retroorbitally. Unfortunately, relatively few permanent uveal melanoma cell lines are available for research and there has been some substantial misidentification [97, 98]. Particularly, C918 and other cell lines are most likely derived from cutaneous but uveal origin [98, 99]. This should be taken into account when interpreting and analyzing past and current data obtained with these cell lines. Further, the diverse molecular landscape of human uveal melanoma can barely be reflected by permanent cell lines. However, recently, novel established permanent cell lines from primary and metastatic uveal melanomas exhibiting a characteristic genetic profile (including GNAQ, GNA11, or BAP1 mutations) allow for further investigations on genetic pathways and their influence on tumor progression and metastasis [100]. Selecting a cell line which phenotypically and genetically reflects desired characteristics is of high importance.

Niederkorn and coworkers investigated multiple human uveal melanoma cell lines in an intraocular model in athymic mice [48–51]. Human xenograft models in immunodeficient mice were frequently used in several studies, for example, on treatment efficacy, on agents or genetic pathways affecting tumor progression, on transcription factors, or on studying immunologic and histologic tumor characteristics [35, 52–58]. Further, labelling human cell lines by bioluminescence allows advanced imaging procedures in the animal. The primary tumor as well as liver and lung metastases are more easily traceable [59–61].

To assess efficacy of treatments on the basis of tumor growth human xenograft models have also been established in nude rats [58], rabbits, and zebrafish. Xenografts were frequently implanted into the eyes of immunosuppressed or nude rabbits with intraocular solid tumor growth, mostly accompanied by metastatic disease, and promising results [62–67]. Rabbits with intraocularly inoculated human uveal melanoma cells were mainly used for basic research [68–71]. Other and upcoming animal models include the injection of human primary and metastatic cell lines into the yolk of zebrafish for screening large libraries of new compounds [72].

Recently, individual patient-derived xenograft (PDX) models have emerged as an important tool for translational research, with the promise of a more personalized approach to patient care. Tumors are obtained from the patient and directly implanted into the model animal. Thus, these models maintain several characteristics of the parental tumor regarding histologic and genetic attributes [101]. First studies with primary and metastatic uveal melanoma transplants into the subcutis or the interscapular fat pad of immunosuppressed mice showed moderate take rates [73–75]. PDX modelling enables tracking of tumor progression and metastasis as well as screening of different (combinatorial) drug strategies to help choose the best and most effective therapy for each individual patient [101, 102]. For uveal melanoma, this approach needs to be further evaluated and established intraocularly in order to achieve methodological advances and increased applicability in research. PDX models of uveal melanoma may help to better understand the complex cascade of uveal melanoma metastasis, to further refine therapy regimes in order to prevent metastasis, or to develop a treatment option once the tumor has metastasized.

However, a major disadvantage of all xenograft animal models is the necessity of immunosuppression. In many malignancies including those derived in immunoprivileged sites like uveal melanoma, tumor progression and the tumor microenvironment are strongly influenced by immune cells [103]. Thus, successful drug screening in xenograft models does not necessarily predict similar effects in humans.

### 2.3. Transgenic Mice

During the last 30 years novel technologies specifically modifying the genome allowed for the generation of transgenic mouse strains. Several criteria have been suggested for such models; for example, mice must carry the same mutation that occurs in the human tumor and mutations should be engineered within the endogenous locus [104]. Such genetically engineered mice are now considered ideal tools to study molecular and genetic pathways in cancer and other diseases. However, these techniques also can be used to generate mouse strains which spontaneously develop certain forms of malignancies. For skin melanoma, for example, RET transgenic mice with spontaneous melanomas are available since 1998 [105]. In the meantime, numerous transgenic mouse models of cutaneous melanoma have been established which closely resembles human skin malignant melanoma with regard to etiology, histopathology, and clinical progression [96].

For uveal melanoma, unfortunately, no such transgenic mouse model is available to date. Many attempts have been undertaken and numerous transgenic skin melanoma models have been investigated for the proliferation of ocular melanocytes. However, in some models no melanocytic proliferation was observed [76]; in others, pigmented intraocular tumors arising in transgenic mice were identified to be of retinal pigment epithelium origin [77, 78] or the small uveal tumors failed to metastasize to the lungs [79–81]. RET.AAD transgenic mice exhibit hyperplastic lesions within all melanocyte-containing sites (skin, eye, meninges, etc.) with early tumor cell dissemination to local and distant organs [82]. As initial melanocytic lesions are mostly found within the uvea, this model was used for investigations on early local and distant tumor growth as well as dissemination [83, 84]. Schiffner and coworkers described a Tg(Grm1) transgenic mouse breed, with spontaneous skin melanoma, which exhibited pigmented choroidal proliferation mimicking spontaneous uveal melanoma [85]. However, applicability as a model for studying intraocular melanoma remains questionable and further studies need to be awaited. Overall, most attempts of finding primary and metastatic uveal melanoma in models of cutaneous melanoma were unsuccessful.

Recently, a GNAQ mutated mouse strain was described which showed neoplastic proliferation not only in choroidal structures but also in dermal nevi and other melanocytic neoplasms. Furthermore and more importantly, a vast majority of these mice exhibited distant metastasis, though exclusively in the lungs [86]. This breed represents the first transgenic mouse model of uveal melanocytic proliferation which is driven by a GNAQ gene alteration. By this means it genetically resembles human uveal melanomas, as about 80% of patients carry a G-protein (GNAQ and/or GNA11) mutation as an early event in tumorigenesis [106, 107]. This may be a first basis for a transgenic mouse model of uveal melanoma and further results need to be awaited. To our knowledge, a new animal model of spontaneous uveal melanoma was established in transgenic zebrafish. Oncogenic resemblance with human uveal melanoma is given as uveal tumorigenesis is driven by an inserted plasmid with a* mitfa*:GNA11 Q209L overexpression (Rose, unpublished data). Publication of this new transgenic model needs to be awaited.

### 2.4. Models of Metastatic Uveal Melanoma

Between 10 and 40% of uveal melanoma patients develop metastatic disease within 10 years after the initial diagnosis [108–110]. Metastases disseminate predominantly hematogenously to the liver and rarely to the lungs or other organs [111]. Liver metastases occur in 95% of patients with metastatic uveal melanoma and result in death in almost all cases [112, 113]. Thus, liver metastases represent a main focus in research. Early detection of uveal melanoma reduces the risk of metastasis and can be lifesaving [114]. Currently, no effective treatment for hepatic or other metastases is available; thus, patients' prognosis worsens dramatically when metastatic disease occurs [108]. As no liver metastasizing primary uveal melanoma model has been established yet, designing a suitable animal model of liver metastasis represents an additional challenge. Different approaches of liver metastatic tumor cell application exist, direct intrahepatic dissemination, splenic implantation with following hematogenous dissemination into the liver, or direct intravenous/intracardiac injection with hematogenous dissemination. By this means the tumor cells reach the liver directly or gain access via the blood stream in order to proliferate at this secondary site. Primary human uveal melanoma cells can be injected into immunosuppressed mice resulting in metastatic disease in most cases [49–51, 59, 60, 90–92] and into immunosuppressed rabbits [64, 67]. On the other hand, murine cutaneous melanoma cells can be orthotopically inoculated into immunocompetent mice [37, 115]. Further, ocular injection of B16LS9 cutaneous melanoma cells leads to hepatic and lung metastases; thus this model mimics the metastatic process from an ocular tumor to secondary sites [20, 33].

However, the generation of metastatic cell clones within a primary tumor requires genetic alterations and subsequent selection of such clones is heavily influenced by interactions with the surrounding microenvironment. Thus, when modelling hepatic metastasis, cell lines generated from a confirmed metastatic origin represent a more appropriate option than cells from the primary ocular tumor. Such models have been investigated and applied for studies in immunodeficient mice [50, 59, 61, 90, 93]. New approaches also use zebrafish or chick embryos for studies on the metastatic behavior of human metastatic uveal melanoma cell lines [72, 94]. Since some uveal “metastatic” cell lines which were thought to originate from metastases turned out to be most likely of primary origin [97], obtained data with these cell lines needs to be interpreted carefully.

Overall, these animal models of metastasis may offer a more detailed investigation of the biological behavior of metastatic uveal melanoma cells in the liver or allow for screening of novel antimetastatic compounds [93]. According to a specific research aspect the adequate cell line and model animal need to be carefully selected. However, a potent model which resembles the dissemination process from an intraocular uveal melanoma into the blood stream in an immunocompetent animal is still missing.

### 2.5. Induced Models

Animals may characteristically develop neoplastic proliferation or tumors after exposure to a given carcinogen or a cancer-causing agent. The agent may be of chemical, radiational, physical, or biological origin and the impact may result in alterations and mutations that lead to uncontrolled cell growth. Certain intraocularly injected oncogenic viruses are capable of inducing neoplasms including melanomas [87, 88]. Two-stage carcinogenesis by chemicals or radiation in pigmented rabbits [89] and other early attempts of induced ocular melanoma resulted in intraocular tumors but did not lead to reproducible animal models [18]. Due to uncontrolled and undirected carcinogenesis this approach barely offers controllability and reproducibility and subsequently does not qualify as a useful animal model. However, treating transgenic mice which harbor a predisposing genetic alteration in an oncogene responsible for uveal melanocytic proliferation might provide an opportunity of a new animal model. By intraocular application of a carcinogenic agent like 7,12-dimethylbenz[a]anthracene (DMBA) uveal tumorigenesis might be accelerated in a controlled manner. Such models already exist for other tumor entities like cutaneous melanoma [116] but have not been examined with respect to uveal melanoma, yet.

## 3. Conclusions

The development of animal models that recapitulate characteristics of human cancers and their clinical response to therapy are a major prerequisite for efficient bench-to-bedside translation and improvement of patients' prognosis, which overall is currently dismal. Research in this area for uveal melanoma has been seriously hampered by a lack of potent experimental* in vivo* models. Unlike other tumor entities, to date, all existing animal models of uveal melanoma exhibit limitations. However, these models represent the best available* in vivo* options and each model has its advantages, which may render it more suitable to address a respective scientific question (Table 1). In essence, syngeneic models suit best for immunologic and tumor biology aspects whereas human xenograft models are commonly used for evaluating treatment strategies. Most importantly, many efforts have been made on establishing transgenic mouse models of spontaneous and metastasizing uveal melanoma which recently provided first promising results.

These limitations in the availability of an integral animal model of uveal melanoma may have fundamentally contributed to delayed research progress. Despite enhanced and refined treatment procedures of the primary tumor, unfortunately, patients' prognosis has not improved significantly since the 1970s [8]. To move forward, it is necessary to better understand and adequately model the unique characteristics of uveal melanoma. Besides genetic attributes, this includes specific features of the ocular immune system leading to a characteristic intraocular tumor microenvironment, the hematogenous dissemination, and colonisation of the liver as well as finally dormancy and the angiogenic switch of hepatic micrometastases. Prevention of metastasis will be the key to improved prognosis. Basic research needs to further focus on the intraocular tumor characteristics and metastatic process of uveal melanoma in order to successfully generate a powerful animal model. This may lead to accelerated research progress on new therapeutic targets. Meanwhile, a better understanding of underlying genetic and molecular abnormalities of uveal melanoma may provide a great opportunity for further development of targeted and individualized therapy regimes in order to improve the prognosis of patients with metastatic disease.

Recent advances in immunotherapy have been followed by a large number of clinical trials in different tumor entities. These new therapeutic strategies are now also being tested in uveal melanoma patients. However, many of these trials are based on results obtained from models of or patients with cutaneous melanoma or other tumor entities. The agents have rarely been tested in animal models of uveal melanoma because a powerful model does not exist and in the case of immunotherapeutics preclinical safety testing was accomplished earlier in other tumor entities. Hence, clinical efficacy of such new therapeutic strategies in uveal melanoma patients might be very variable or even disappointing.

Overall, in order to achieve an improvement in patients' outcomes a better understanding of the pathogenesis of uveal melanoma is required which may be accomplished by using effective* in vitro* methods like 3D tumor cultures or powerful animal models of intraocular melanoma. Established models may be further refined (improved injection techniques, authenticated or new cell lines) and based on existing limitations they need to be carefully selected for a respective scientific question. Basic research may further focus on the generation and establishment of a transgenic animal model as this type of model offers strong advantages regarding immunologic, genetic, and histopathologic aspects. To reliably test novel therapeutic regimes and accurately identify therapy responses a personalized approach seems to be most promising. Therefore, PDX models for testing compounds or combinatorial therapy regimes (including targeted gene therapy and immunotherapy) may offer the best option. Further research on this type of model is strongly needed in uveal melanoma. Hence, in future these different animal models should be the basis for both biological and pharmacological testing and for rational clinical trials, thereby guiding treatment decisions and eventually improving the prognosis in patients with uveal melanoma.

## Figures and Tables

**Table 1:** Animal models of uveal melanoma.

	Strength	Limitation	Comment	References
Intraocular injection of syngeneic cutaneous melanoma cells	Intraocular melanoma in an immunocompetent animal, reliable reproducibility	Different genetic background, difficulties to achieve hematogenously spread metastasis	Qualifies for studies on the microenvironment (immunologic or angiogenic aspects)	In mice, [20–37] In rabbits, [18, 42–45]

Intraocular injection of human uveal melanoma cells	Human uveal melanoma: its progression and behavior can be investigated	Necessity of immunosuppression, equivocal permanent cell lines	Frequently used for evaluating treatment options or screening therapeutic agents	In mice, [35, 48–61] In rats, [58] In rabbits, [62–71] In zebrafish, [72]

Patient-derived xenografts (PDX)	Individualized investigation of tumor progression and screening of therapeutic compounds	Necessity of immunosuppression, fresh material not constantly available for research	To date, only preliminary studies for uveal melanoma, further research and refinement needed	In mice, [73–75]

Transgenic models of cutaneous melanoma	Spontaneous uveal proliferation in an immunocompetent animal	Different genetic background, no reliable metastasis	Spontaneous skin melanoma does not necessarily guarantee uveal proliferations	In mice, [76–85]

Transgenic models of uveal melanoma	Spontaneous uveal melanoma in an immunocompetent animal with a genetic background similar to human uveal melanoma	No reliable hematogenous metastasis to the liver yet	Promising basis which demands further research	In mice, [86] In zebrafish, [Rose, unpublished]

Induced models	Easy to induce	In wild type animals uncontrolled, undirected tumorigenesis	If performed in transgenic animals potentially a promising approach	Viruses, [87, 88] Chemicals/radiation [89], reviewed in [18]

Models of liver metastasis	Investigation of behavior of metastatic uveal/cutaneous melanoma cells	No “true” metastatic process from a primary tumor, partially in immunosuppressed animals	If using metastatic cell lines, screening of novel antimetastatic compounds is possible	Primary human cell lines in mice, [49–51, 59, 60, 90–92] Primary human cell lines in rabbits, [64, 67] Metastatic human cell lines in mice, [50, 59, 61, 90, 93] Metastatic human cell lines in others, [72, 94]
